# Piloting ‘Virtual Ward’: a novel platform for delivering medical student education by residents

**DOI:** 10.1186/s12909-022-03465-w

**Published:** 2022-05-21

**Authors:** Coralea Kappel, Waseem Hijazi, Nishma Singhal

**Affiliations:** 1 Department of Internal Medicine, McMaster University, Hamilton, ON Canada; 2 Department of Cardiac Sciences, University of Calgary, Calgary, AB Canada; 3 Division of Infectious Diseases, Department of Medicine, McMaster University, Hamilton, ON Canada

**Keywords:** Virtual teaching, Medical student, Medical education, COVID-19

## Abstract

**Background:**

Clinical experiences lie at the heart of undergraduate medical education (UGME). COVID-19 related disruptions in Medical Education impacted medical students substantially. As educators, efforts directed at developing new mediums to educate our medical students in the face of these new limitations were vital. The Virtual Ward (VW) pilot was an inaugural resident-driven, virtual educational opportunity aimed at supplement the learning of core internal medicine skills for undergraduate medical students.

**Methods:**

Interested medical students were paired in groups of 5–6 with an internal medicine resident tutor. The McMaster University UGME core internal medicine topic list was provided to resident tutors to teach in an open, morning-report format in which students directed content selection. Following completion of the VW series, we distributed an online anonymous survey using a 5-point Likert scale to gauge the efficacy of the intervention and compare it to existing learning modalities offered by the UGME.

**Results:**

In total, 166 medical students and 27 internal medicine resident tutors participated in the VW pilot. 46 (28%) medical students responded to the survey and 96% of survey respondents rated the sessions as being helpful to their learning. The majority rated VW superior to existing learning modalities and 94% thought VW should continue after COVID-related restrictions abate.

**Conclusions:**

VW is a novel educational platform that was very well received by learners. We propose VW may have a continued supplemental role post-pandemic to help with translation of knowledge to clinical skills and provide an additional avenue of mentorship for students.

**Supplementary information:**

The online version contains supplementary material available at 10.1186/s12909-022-03465-w.

## Background

As a result of the COVID-19 pandemic, medical student education was significantly affected. In our institution, medical students were pulled from direct clinical activities in March of 2020 for four months, their education becoming largely module-based. Opportunities for direct clinical experiences were limited thereby delaying the development of clinical problem-solving acumen. Importantly, medical students were also disconnected from their resident near-peers, which research has identified as important for mentorship and education due to enhanced social and cognitive congruence [1]. In response to these educational challenges, VW was developed based on the principles of near-peer education and case-based learning by virtually connecting internal medicine (IM) resident tutors with pre-clerkship or clinical clerk medical students.

Peer-education, first proposed in 1988 by Whitman and Fife, is a widely-used teaching model that is based on social congruence, referring to the approachability of peer-educators given their similarity to the capabilities of learners [2]. Peer-education improves learners’ understanding of subject matter, provides a ‘safer’ learning environment, augments learner confidence and enhances motivation. Furthermore, it has been shown that students give more value to an activity when it aligns with their own objectives and expectations [3]. Therefore, student motivation emerges when teaching sessions are designed to explore and define these [4]. Case-Based Learning (CBL) is another effective learning method that exposes students to real-world scenarios that need to be solved using their own skills and knowledge [5]. This model of learning supports flexibility and a collaborative learning style that is effective at developing critical thinking skills and integrating problem-solving into practice [6, 7]. Early in the pandemic, VW served as an opportunity for students to remain connected to resident near-peers and benefit from their ancillary teaching. However, given the novelty of VW as a learning tool that combines these effective learning methods, we sought to investigate its construct validity utilizing a post-survey method.

## Methods

In the initial phase, we surveyed interest in VW by sending out an email to medical students describing the initiative and linking a Google Forms survey. This survey gauged preliminary interest and determined which topics or skills students wished to develop related to internal medicine (see Additional file 1: Appendix B). After noting overwhelming interest from McMaster University medical students in the initial survey, specifically 151 students, we reached out to the UGME program regarding formally launching the VW initiative. After the UGME program expressed interest, their leadership team provided us with a mailing list of current medical students. Two seperate sign-up tools were sent out: one to preclerkship students and another to clerkship students. A formal Virtual Ward sign-up tool was sent to the preclerkship students via Google Forms, asking them to specify their level of training, the desired frequency of sessions (i.e. weekly, biweekly or monthly) and a list of three primary learning goals. In the sign-up email, we requested that each student commit to a minimum of three VW sessions over 6 weeks but beyond that, the overall duration of VW and session frequency was left open to each group. For clerkship students, the Internal Medicine Clerkship Coordinator sent a sign-up form with identical survey items at the start of their internal medicine clerkship rotation. This group was asked to complete a minimum of three VW sessions over the course of their 4-week internal medicine block, but some groups extended beyond the minimum three session requirement. We then recruited volunteer IM resident tutors across postgraduate years one to three (PGY1 to PGY3). In total, we recruited a team of 46 interested internal medicine resident tutors. Based on availability, 27 internal medicine residents were paired with a total of 166 interested medical students (51 clerks and 155 pre-clerks) in groups of 5–6.

**Fig.1:**
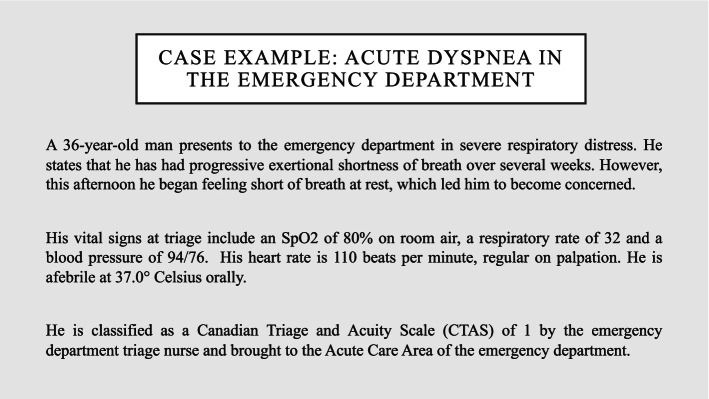
Example of case vignette

To provide a foundation for the teaching sessions, resident tutors were given guidance regarding the expected case-based format, a template powerpoint to guide their sessions (Additional file 1: Appendix A) and a list titled 'Essential Clinical Experiences in Internal Medicine' provided by the McMaster UGME (Additional file 1: Appendix B). The online platform for these sessions was Zoom, which was accessible through the University with unlimited session duration and free of cost. Although each group had flexibility in their style, sessions began with cased-based descriptions of a patient (Fig. 1). Students would first be asked to elicit history and physical examination details and then be offered results. Later on, diagnostic tests such as pulmonary function tests, serology, imaging and electrocardiograms would be presented and become subject to interpretation. In general, students would request information from the tutor and be provided with additional clinical information in an iterative process, similar to morning report-type sessions. Utilizing problem-solving skills to identify the next appropriate diagnostic and therapeutic steps, as well as to develop a differential diagnosis, was key. The sessions also afforded an opportunity for medical students to network with IM residents and develop a mentorship relationship. Based on shared learning goals, students subsequently determined the next session’s topic.

In terms of resident preparation, beyond being given a powerpoint template and a list of UGME educational priorities, residents accessed educational materials from the IM program Academic Half-Day Curriculum and other evidence-based resources such as JAMA Rational Clinical Exam and UpToDate to provide materials. Clinical skill resources such as Bate’s Guide to Physical Examination and History Taking were used for cases that involved detailed physical examination discussions. Ultimately, each resident drew upon a separate set of resources to prepare a well-researched case. A Google Drive folder was created for residents to share cases among each other. Residents prepared cases from an array of disciplines, including hematology, respirology, cardiology, critical care, infectious disease, and others. Although cases could include all components from pathophysiology, therapeutics, diagnostic and laboratory data interpretation, certain cases focused on one or more of these in heightened detail.

Upon completion of the VW sessions, we asked resident tutors to distribute a Google Forms survey link to their respective groups. This consisted of an online anonymous quantitative survey using a 5-point Likert scale. Students were asked to specify how many sessions they participated in and compare their learning experience to existing learning modalities offered by the UGME program. In addition, students were given the option of leaving comments describing their experience and suggestions for improvement and if the initiative should continue post-pandemic. The goal was to determine the value of VW and its value as a complementary tool to the existing UGME learning experiences in the short and long-term. More broadly, we aimed to understand its role as a future curricular tool for medical students during, and beyond, the restricted learning opportunities in the context of the COVID-19 pandemic.

## Results

In the initial survey of interest, 100% of 151 students respondents expressed interest in these sessions. With regards to session frequency, 35.8% (54 students) stated they preferred a session every two weeks and 33.8% (51 students) preferred weekly sessions. From a list of potential topics, the students were most interested in learning and developing skills in the interpretation of electrocardiograms (ECGs), laboratory data, and diagnostic imaging (i.e., chest and abdominal x-rays), as well as problem solving skills in general. Ultimately, 166 medical students registered for VW.

A total of 46 (28%) medical students responded to the post-survey; 38 (83%) pre-clerks and 8 (17%) clerks. Each group conducted a median of five sessions (range: 2–9 sessions). In terms of topics that students felt more confident with following the sessions: 89% said they were more confident in developing a differential diagnosis, 76% managing common internal medicine issues, 58% interpreting laboratory data, 56% taking histories, 42% interpreting ECGs/arterial blood gases and 36% felt more comfortable interpreting imaging data.

**Fig.2:**
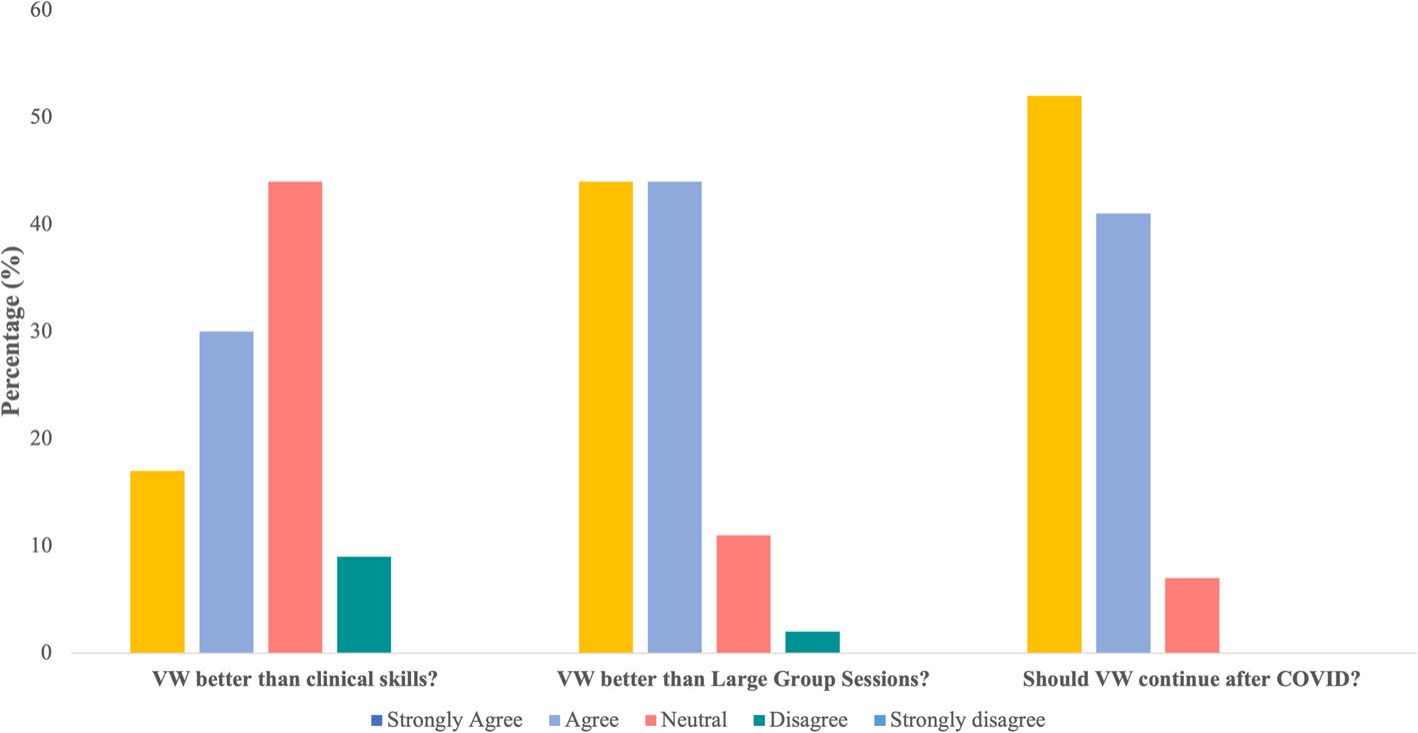
Student perception of Virtual Ward compared to existing curricular components

Thirty three (72%) students rated their overall learning experience as extremely helpful, and 11 (24%) students rated it as helpful. Following VW, 41 (89%) students agreed that they felt more prepared for clinical encounters on the ward. In the absence of medical school curriculum disruptions from COVID-19, 43 (93%) students agreed that VW would still be a beneficial complementary learning tool (Fig. 2). Forty four (96%) students believed that the preclerkship Integration Foundation Unit of the McMaster UGME program would be the most useful time to have VW sessions.

In the free text section of the post-survey, common themes emerged. Namely, students' learning experience (i.e., helpful, easy approaches, engaging) and quality of the resident facilitators’ teaching skills (i.e., great teachers, excellent mentorship, interactive, well-prepared). Students frequently commented that VW was flexible and tailored to their needs and interests.

## Discussion

In light of pandemic-related disruptions to undergraduate medical education, a low-cost, resident-driven intervention demonstrated excellent face validity in delivering internal medicine education to preclerkship and clerkship students. As judged by students, it was at least comparable to existing medical school teaching modalities in delivering content, especially compared to large-group sessions (i.e., lectures) and clinical skills sessions. There was strong agreement that Virtual Ward should remain an educational opportunity after pandemic-related restrictions are lifted in addition to traditional curricular components. Additionally, Virtual Ward was easy to administer and came at no extra cost to the university or students.

For internal medicine residents, Virtual Ward was an opportunity to remain connected with the medical community and develop their skills as a clinician-educator. In addition, camaraderie was developed between medical students and residents within their local sphere, a factor identified as important for the success of educational initiatives [1]. Worldwide virtual learning experiences have been rapidly and broadly adopted as part of the COVID-19 modified experiences in medical programs [7, 8]. Past studies have validated near-peer teaching as an effective modality for delivering education, with a predominantly positive effect on residents and tutors [9, 10]. One rationale supporting this positive dyad is the concept of cognitive congruence, termed by Lockspeiser et al. [11], which helps students to feel more at ease with a near-peer, who conceivably would appreciate their local educational goals more closely [12, 13].

Student participation in VW went beyond the educational component. Students commented that they value the mentorship opportunities that arose from interacting with residents in small-group sessions, especially since such residents were proximal to their stage of training. Some of these mentorship-mentee relationships continued beyond the VW tutorials and ultimately function as an ongoing source of career and scholastic advice. In addition, near-peer relationships between students and residents are more likely to uncover aspects of the ‘hidden curriculum’ and may have a unique benefit for students from disadvantaged backgrounds [14, 15].

At our institution, even with the return of clerks to the ward, there remains a paucity of clinical experiences that VW could conceivably fill. Clerks continue to have restricted hours on the clinical teaching units, thus VW could supplement some of those displaced learning opportunities. Pre-clerks may also stand to benefit from engaging with residents at a time when they may not have significant prior clinical exposure on the wards. The morning report and open format also allows flexibility to meet individual learning needs while fostering clinical reasoning skills. Given the large proportion of medical students supporting this initiative beyond the COVID-19 pandemic, we believe there is a need to explore VW as a curricular component in the UGME program.

The pandemic has highlighted that educational modalities require constant evolution with changes in policy and social distancing. Our post survey for Virtual Ward showed that many students saw a role for these additional resident-led peer-to-peer sessions even in the post-pandemic world when normal educational activities resume. For example, 95% of our respondents felt it could be added as a mandatory teaching activity in the Integration Foundation Unit of McMaster's medical school preclerkship curriculum. Most students felt these sessions were more effective or equally effective as existing pre-pandemic educational tools. We believe this provides face validity that these virtual sessions have merit and can continue post-pandemic; we have liaised with the UGME leadership team to advocate for this. In addition, the sessions could transition to become face to face should that be a strong desire among students. We believe this publication will serve as an impetus for the UGME to further consider implementing the program and generate discussions about new and innovating ways of teaching.

One limitation of this analysis is the relatively low response rate to the post-intervention survey, underscoring the possibility that our results depict a subset of students who had a positive experience. However, for an internal survey, our response rate of 28% is within the average range typically reported. Additionally, even if respondents were biased, a sufficient proportion of participants indicated that the VW initiative is highly worthwhile and that the program exploration. Another limitation is that in these early stages of the project, we have not established correlation of VW sessions with validated criterions for performance, such as in-training evaluation reports or formal evaluations like exam results and performance progress index. Obtaining information regarding students’ increased confidence with respect to specific topics within the UGME list could be an avenue of research in the future.

## Conclusions

In summary, through a real-life roll out of a virtual Zoom-based, resident-run and freestyle internal medicine tutorial program, we demonstrated that students found Virtual Ward to be more or equally effective as traditional learning modalities. We propose that this low-cost, highly accessible model can be emulated and implemented in various other educational contexts to support medical student education. At our Institution, similar efforts are being considered to include educational and mentorship opportunities.

## Supplementary information

**Additional file 1.**

## Data Availability

The datasets used and/or analyzed during the current study are available from the corresponding author on reasonable request. Given the lack of funding for this project, the data is not available on a public repository. However, the data can be made available by corresponding author on a reasonable request.
