# Supplementary figures and images for: Piloting ‘Virtual Ward’: a novel platform for delivering medical student education by residents
Source: BMC Med Educ. 2022 May 21;22:392. doi: 10.1186/s12909-022-03465-w (PMC9123921; doi:10.1186/s12909-022-03465-w)

**Appendix A**

**Appendix B**
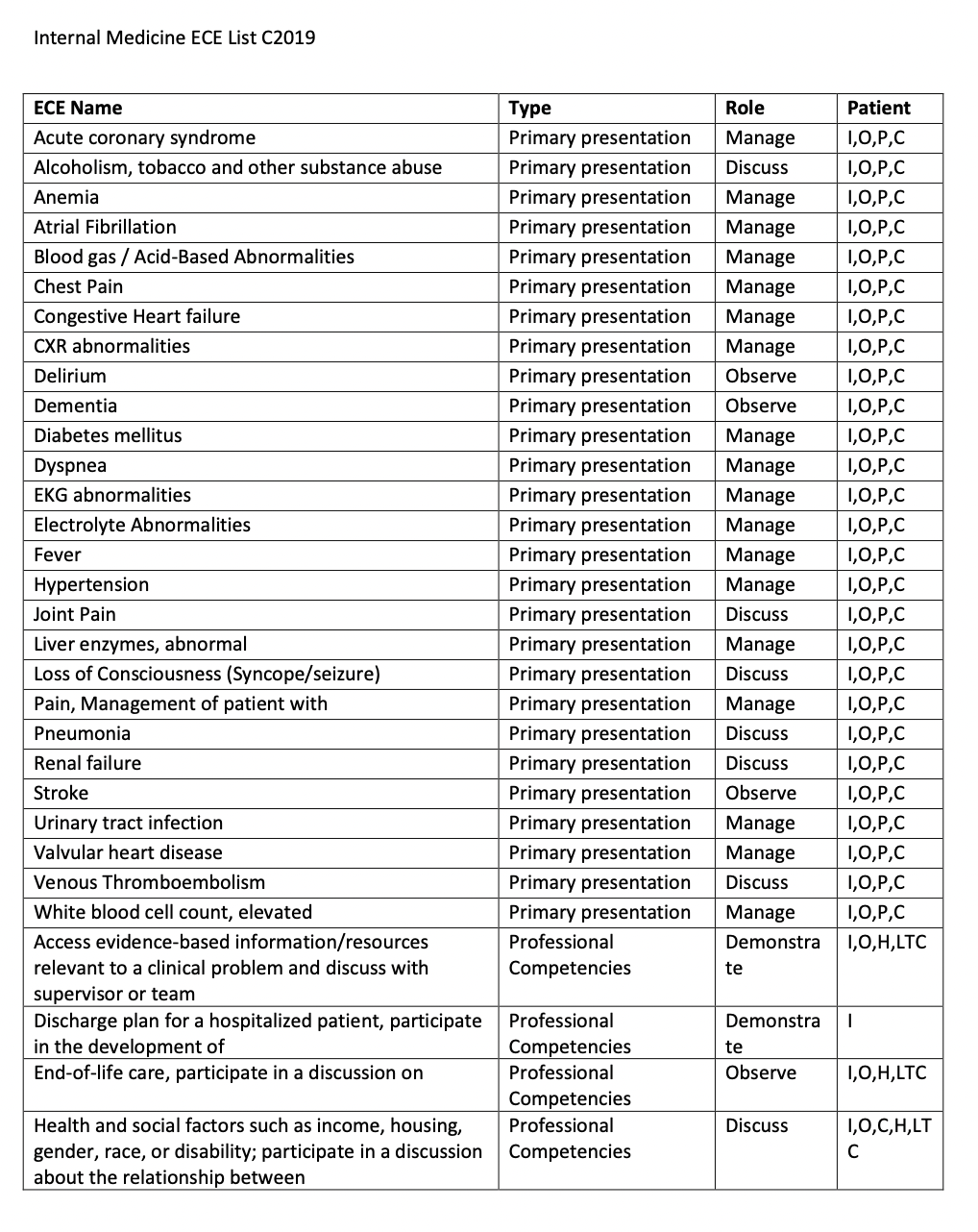

Supplement: Supplementary file 1 — Additional file 1. [file 12909_2022_3465_MOESM1_ESM.docx]
